# High *versus* low mean arterial pressure target in liver transplant patients. An open, controlled, single-center, randomized clinical trial - Protocol and methods (LIVER-PAM)

**DOI:** 10.5935/2965-2774.20230336-en

**Published:** 2023

**Authors:** Rodolpho Augusto de Moura Pedro, Bruna Carla Scharranch, Lucas de Oliveira Araújo, Luciana Severo Brandão, Lúcia da Conceição Andrade, Wellington Andraus, Luís Augusto Carneiro D’Albuquerque, Luíz Marcelo Sá Malbouisson

**Affiliations:** 1 Department of Gastroenterology, Hospital das Clínicas, Faculdade de Medicina, Universidade de São Paulo - São Paulo (SP), Brazil; 2 Department of Nephrology, Hospital das Clínicas, Faculdade de Medicina, Universidade de São Paulo - São Paulo (SP), Brazil; 3 Discipline of Anesthesiology, Department of Surgery, Hospital das Clínicas, Faculdade de Medicina de São Paulo - São Paulo (SP), Brazil

**Keywords:** Arterial pressure, Renal insufficiency, Liver transplantation, Postoperative period, Perioperative care, Hemodynamics

## Abstract

**Objective:**

To explain the rationale and protocol of the methods and analyses to be used in the LIVER-PAM randomized clinical trial, which seeks to understand whether a higher mean arterial pressure is capable of reducing the incidence of renal dysfunction postoperatively after liver transplantation.

**Methods:**

LIVER-PAM is an open-label, randomized, controlled, singlecenter clinical trial. Patients randomized to the intervention group will have a mean arterial pressure of 85 - 90mmHg in the initial 24 hours of postoperative management, while patients in the control group will have a mean arterial pressure of 65 - 70mmHg in the same period. A sample of 174 patients will be required to demonstrate a 20% reduction in the absolute incidence of renal dysfunction, with a power of 80% and an alpha of 0.05.

**Conclusion:**

If a 20% reduction in the absolute incidence of renal dysfunction in the postoperative period of liver transplantation is achieved with higher target mean arterial pressure in the first 24 hours, this would represent an inexpensive and simple therapy for improving current outcomes in the management of liver transplant patients.

**ClinicalTrials.gov Registry:** NCT05068713

## INTRODUCTION

Postoperative patient management after liver transplantation is complex and challenging, as there is an increased risk to the viability of the graft and to the survival of the patient.^(1)^ In this scenario, hemodynamic optimization may be a determining factor in the evolution of the clinical condition.

Renal dysfunction is one of the most frequent postoperative complications after liver transplantation and is considered the most important predictor of poor long-term outcome,^(2)^ as it is associated with higher mortality^(3)^ and impacts the quality of life, as 18.1% of these patients will have a renal clearance < 30mL/minute/1.73m^2^ within 5 years.^(4)^ This dysfunction is related to a combination of preoperative (previous renal function, hepatorenal syndrome and diuretics), intraoperative (ischemia‒reperfusion injury, shock and bleeding) and postoperative events (nephrotoxic drugs, water balance and hemodynamic optimization).^(4)^

One of the classical drivers of renal dysfunction is hypoperfusion, which is closely linked to mean arterial pressure (MAP).^(5)^ The classic target of MAP > 65mmHg is described as the safety threshold,^(6,7)^ although a trial employing higher MAP levels showed lower renal dysfunction in certain subgroups of patients with sepsis.^(8)^ In surgical patients, hypotension is associated with a higher incidence of acute renal dysfunction, and this risk increases with the length of the insult.^(9,10)^ A reduction in other postoperative complications has been observed following hemodynamic optimization for achieving a MAP closer to the patient’s baseline.^(11)^

In the specific scenario of liver transplantation, retrospective data adjusted for the propensity score inferred the best renal outcome with a MAP > 90mmHg,^(12)^ while a physiological evaluation indicated that a reduction in renal blood flow occurs below a MAP threshold of 75mmHg.^(13)^

Preventing renal dysfunction in the postoperative period may represent an important way to improve short- and long-term patient outcomes, with blood pressure being a possible therapeutic target. However, there is no definition of the best blood pressure target and the exact relationship between different blood pressure levels and renal outcome. In addition, there are currently few randomized clinical trials in liver transplant intensive care, during which renal dysfunction is more frequent.^(12)^ Considering the current low availability of organs due to the high number of candidate recipients,^(14)^ understanding tools that may yield better results and reduce complications and expenses is a priority.

Our hypothesis is that pressure optimization in the first 24 hours after surgery with higher MAP levels (85 - 90mmHg) is superior to usual care (MAP 65 - 70mmHg) in preventing acute renal failure in the first 7 days after liver transplantation surgery.

## METHODS

### Study design

LIVER-PAM will be a single-center, prospective, randomized, open-label, controlled study in which patients will be randomized to the intervention group with the highest MAP (85 - 90mmHg) or the control group with the lowest MAP (65 - 70mmHg) for a period of 24 hours after admission to the intensive care unit (ICU) for liver transplantation. The study will be conducted in the ICU of the Department of Gastroenterology, Hospital das Clínicas, School of Medicine, University of São Paulo (USP). The authors reinforce their commitment to following the guidelines for the reporting of randomized clinical trials (CONSORT), the international registry of trials and institutional ethical approval. The study is registered on ClinicalTrials.gov under identification number NCT05068713, and the study was approved by the Research Ethics Committee (REC) of the institution under opinion 4,887,829, referring to the second version of the protocol on September 16, 2022.

### Main objective

The primary goal of the study will be to evaluate whether higher (85 - 90mmHg) *versus* usual MAP levels (65 - 70mmHg) in the first 24 hours after liver transplantation are associated with a reduced incidence of acute renal dysfunction at 7 days, according to the adaptation of the KDIGO criteria,^(15)^ using a 1.5-fold increase in baseline creatinine as a cutoff point (Appendix 1). Patients who die within the first 7 days will be considered positive for the main outcome measure.

### Secondary objective

To evaluate whether higher (85 - 90mmHg) *versus* usual MAP levels (65 - 70mmHg) in the first 24 hours after liver transplantation surgery are associated with 28-day mortality; retransplantation in 28 days; renal replacement therapy in 7 days; surgical site infection within 7 days; days alive and free of renal dysfunction in 7 days; renal dysfunction according to the values of neutrophil gelatinase-associated lipocalin (NGAL) at admission and after 48 hours; length of stay in the ICU; length of hospital stay; days alive and hospitalization-free in 28 days; liver graft dysfunction (initial poor function subtype) in 7 days; liver graft dysfunction (primary nonfunction) at 10 days and major adverse kidney events at 28 days (MAKE28).

### Other outcomes (safety)

The safety outcomes to be assessed are the incidence of major postoperative bleeding at 7 days, transfusion of blood components at 7 days and incidence of arrhythmias requiring drug or electrical therapy at 7 days.

### Definition of outcomes

The definitions of the outcomes cited above are described in appendix 1 with their respective references.

### Inclusion criteria

All patients older than 18 years of age admitted to the ICU after liver transplantation will be evaluated for randomization. All patients who receive a confirmed offer of an organ for liver transplantation and are referred to the operating room to begin the procedure will be screened for inclusion in the study. At the end of surgery, patients admitted to the ICU and who are eligible according to the criteria described here will be randomized within 2 hours of admission.

### Exclusion criteria

The exclusion criteria were defined with the goals of safety and limiting confounders, as follows: liver transplantation due to fulminant hepatitis; liver-kidney transplants; hepatorenal syndrome being treated within 48 hours pretransplantation; renal replacement therapy in the 15 days prior to transplantation; persistent refractory shock intraoperatively or on ICU admission (noradrenaline > 1 mcg/kg/minute associated with a second vasopressor for more than 2 hours); cardiorespiratory arrest intraoperatively or on admission; refusal of the physician responsible for the care; contraindication to the prolonged use of vasopressors; pregnancy; and retransplantation at an interval of less than 6 months.

Patients or family members will be informed of their right to interrupt the intervention if they wish to withdraw from participation at any stage of the study. The team of professionals involved in the care of these patients will be informed of their rights to interrupt the protocol according to clinical judgment or in the event of a serious adverse event attributed to the intervention.

### Randomization and blinding

Patients will be randomized within 2 hours of ICU admission after liver transplantation using a simple 1:1 allocation sequence without group stratification. The randomization will be performed electronically by the randomization tool of the Redcap® system. The randomization sequence will be generated *online* and entered into the system by a staff member who will not participate in the participant tracking process, in obtaining informed consent from the patient or in the clinical management of the cases. The researcher will also not participate in the evaluation of the study outcome data in order to ensure compliance with the allocation concealment process.

As the interventions in both groups are based on target MAP levels to be followed by the professionals involved in the care, the authors chose not to blind the interventions, which, although possible, would be complex to perform in the center in question. However, the professional responsible for collecting the outcome data will be an external research nurse, with no affiliation with the institution where the study is conducted and who is not involved with the care offered to each patient. The same care will be taken to hire the statistician who will analyze the results, ensuring that he or she will not be aware of the allocated groups. To minimize the risk of bias, an objective primary outcome was chosen based on test results measured by a certified laboratory.

### Intervention

#### General management (both groups)

All patients will be treated according to the current care protocol of the institution, which aims to achieve normal oxygenation (oxygen saturation - SpO2 ≥ 94%), hemoglobin (≥ 7g/dL), body temperature (≥ 35ºC) and cardiac index (> 2.5L/minute/m^2^) levels. Professionals on duty not participating in the study will determine fluid infusion based on clinical criteria including heart rate, cardiac index, blood pressure, urine output, core-peripheral temperature gradient, serum lactate level and base excess. The MAP will be determined according to the allocated group until 24 hours after admission to the ICU have passed. The use of antibiotic prophylaxis will be defined by the team of transplant infectiologists before patient randomization; the initial and subsequent immunosuppressive doses will be defined by the surgical team according to the institutional protocol. The hemodynamic variables will be monitored by means of an invasive pressure catheter, pulmonary artery catheter and/or ultrasonography. Our priority is to construct a pragmatic protocol that could increase adherence to the trial. Given that the study setting is a public ICU in a low/middle income country with limited human resources,^(16)^ the heterogeneity of the actors involved and the widespread number of admissions for transplants, the indications for fluids, the type of fluid and the rate to be infused will be left to the discretion of the attending physician; these data will be collected and described in the results. The discretion of the attending physician also extends to the use of inotropes because although the institutional practice is to use such inotropes only when the cardiac index is below the reference value mentioned above, this practice requires individualization in cases of portopulmonary hypertension, in that the use of milrinone may be requested individually.

#### Specific management of the intervention group

The intervention will begin upon admission to the intensive care unit and will continue for 24 hours after arrival at the ICU. The MAP target of the intervention group will be 85 - 90mmHg in the first 24 hours after surgery. The MAP will be measured using an invasive blood pressure catheter, measured at 1-hour intervals during the first 12 hours and at 2-hour intervals thereafter until the 24 hours have been completed (according to the protocol of the ICU). Patients with a MAP lower than the established target may receive fluids or vasopressors according to the discretion of the attending physician to achieve the established target MAP.

Noradrenaline will be the initial vasopressor of choice, and the second will be determined by the attending physician. In hypotensive patients with reduced cardiac output as monitored by pulmonary artery catheter or transthoracic echocardiogram, inotropes may be used at the discretion of the assistant team. Patients who naturally present with a MAP > 90 mmHg even after complete weaning from vasopressors will not receive additional vasodilators to reduce the MAP to the study target. The use of vasodilators in hypertensive patients for the purpose of blood pressure control will be determined according to the decision of the attending physician.

#### Specific management of the control group

Patients in the control group will undergo the care regimen described above, the only difference being the target MAP, which in this group will be 65 - 70mmHg during the first 24 postoperative hours. The MAP will be measured using an invasive blood pressure catheter. Patients who naturally present with a MAP > 70mmHg even after complete weaning from vasopressors will not receive vasodilators to reduce the MAP to the target range. The use of vasodilators in hypertensive patients for the purpose of blood pressure control will be determined according to the decision of the attending physician. The MAP will be measured using an invasive blood pressure catheter at the same intervals described for the intervention group.

### Serious or unexpected adverse events

Any adverse events identified to be related to the study intervention, whether serious or unexpected, as assessed by researchers or professionals involved in the care will be immediately reported to the competent authorities, in particular to the management of the unit, the management of the hospital and the responsible REC. The patient will receive all necessary care to reverse or minimize any damage resulting from the study. Given the suspicion of such events, if the patient is in the intervention group, the intervention will be interrupted, and the data will be analyzed according to an intention to treat protocol, except in cases in which the patient or family members request withdrawal of the individual from the study. We believe that safety should be the priority in a randomized clinical trial. The absence of an external committee in our study is the result of extensive discussion in the design of the study, in which the safety of the intervention chosen and the additional costs that the committee would represent to the project were considered. The final decision not to include a monitoring committee was defined, described and sent to the REC responsible for the original project.

### Data collection

The following data will be collected from all patients: name, sex, age, race, height, weight, hospital identification number, date of birth, etiological diagnostic indicator of transplantation, pretransplantation Model for End-stage Liver Disease (MELD) score, origin before transplantation (ICU, ward or home), comorbidities (diabetes mellitus, heart failure, arterial hypertension, nondialytic chronic renal failure, active cancer, previous stroke or transient ischemic attack or immunosuppressive therapy in the last 30 days before surgery), preoperative creatinine, donor risk index (DRI) and use of nephrotoxic drugs in the week before transplantation.

The following intraoperative data will also be collected: anesthesia time, total and cold ischemia time of the liver graft, presence of ischemia‒reperfusion syndrome, time of admission to the ICU, use of blood products, antibiotic prophylaxis, initial value of arterial lactate, peak arterial lactate, final value of arterial lactate, use of basiliximab, use of fluids, type of fluids, episodes of intraoperative hypotension and loss of fluids.

The following postoperative data will also be collected: length of hospital stay after transplantation; length of stay in the ICU; time of mechanical ventilation; duration of vasopressor use and maximum vasopressor dose; need for renal replacement therapy; need for postoperative transfusion; primary graft dysfunction; secondary graft dysfunction; need for retransplantation; surgical site infection; Sequential Organ Failure Assessment (SOFA) score on admission to the ICU; D1, D3 and D7; daily creatinine, to evaluate the presence of acute kidney failure (AKI) in the first 28 days after transplantation or until hospital outcome if this occurs before the described period; admission and 48-hour urinary NGAL; presence of surgical site infection in the first 7 days after transplantation; arrhythmias requiring chemical or electrical treatment in the first 7 days after transplantation; diagnosis of acute coronary syndrome in the first 7 days after transplantation; volume of crystalloids infused in the first 24 hours after surgery; volume of colloids infused in the first 24 hours after surgery; diuresis in the first 24 hours after surgery; drainage output in the first 24 hours after surgery; presence of major bleeding in the first 24 hours after surgery; need for transfusion in the first 24 hours after surgery; antibiotic prophylaxis in the first 24 hours after surgery; use of nephrotoxic drugs in the first 7 days; immunosuppression in the first 7 days and maximum tacrolimus level within 7 days; new surgery within 7 days after randomization; ICU readmission for treatment of complications within 7 days after randomization; need for retransplantation within 28 days and ICU and hospital outcome in 28 days (discharge, hospitalization or death).

Data collection will be performed by an external professional (research nurse) with no connection to the unit. This professional will have access to the data described but will not have any role in the randomization and management of patients participating in the study. Such data will be collected in an electronic form via Redcap® and will remain protected by a nontransferable password. The professional will perform serial evaluations to ensure the follow-up of all patients until the 28th postoperative day. The final result collected will be available, also by means of a password, to the external professional in charge of the statistical calculations, as well as to the main researcher.

### Data handling and record keeping

The confidentiality of the information related to the participants will be protected by the team involved in the conduction of the research project, and the identity of the patients will at no time be revealed, as provided in Resolution 466/12 of the National Health Council, item III.2.i, and other legislation in force. The data will be stored electronically with access protected by a password.

### Sample size calculation

Based on the primary outcome incidence of 73.7% (acute renal dysfunction within 7 days) obtained from a database of the same population and location, 174 patients will be required to demonstrate an absolute reduction of 20% in the primary outcome, with a power statistic of 80% and alpha of 5%. The data used for this calculation were obtained by calculating the incidence of AKI based on an increase in serum creatinine by 1.5 times the baseline value. The criterion used to choose the effect (20% absolute reduction in the AKI outcome) was chosen based on the principle of clinical relevance. All patients selected for transplantation will be evaluated regarding the inclusion and exclusion criteria and will be informed of the study in a standardized way if able to participate.

### Statistical analysis

The analyses will be performed according to the “intention-to-treat” principle; all randomized patients will be included in the analysis according to the treatment group to which they were randomly assigned. Summary statistics per group, with treatment effects, 95% confidence intervals and p values, will be presented for the primary and secondary outcomes. Continuous variables will be tested for normality using the Shapiro‒Wilk test after graphic analysis by histogram. Parametric continuous variables between two groups will be compared with the unpaired *t* test and within the same group with the paired *t* test. If analysis among more than two groups is required, analysis of variance (ANOVA) will be used. Nonparametric continuous variables will be evaluated using the Wilcoxon test or the Mann‒Whitney U test between two groups and the Kruskal‒Wallis test between three or more groups. Categorical variables will be evaluated using the chi-square test or Fisher’s exact test, as appropriate. P values < 0.05 will be considered statistically significant. The outcomes of hospitalization-free days or renal dysfunction over time will be calculated based on the 7- and 28-day follow-up, respectively, using Cox regression models. The calculations of survival curves will be performed with the Kaplan‒Meier method. For the statistical analysis of outcomes, the results will be adjusted for previous renal function, and logistic regression will be used to adjust for other confounding factors that generate inconsistency in the baseline that were not corrected by the randomization of the study (use of nephrotoxic drugs, maximum tacrolimus level in the first week, intraoperative fluid balance and intraoperative major bleeding). No statistical adjustments will be provided for multiple comparisons in the case of secondary outcomes, and therefore, we suggest that these be interpreted as exploratory analyses.

The existence of missing data will be described in the publication and managed with simple exclusion from the analysis. For this study, no interim analyses are foreseen.

### Subgroup analysis

As previously sent to the REC, the following subgroup analyses are planned: present or absent previous renal dysfunction, present or absent liver graft dysfunction, from home or hospitalized, use or nonuse of antibiotics for > 48 hours, serum tacrolimus value > 15 in 7 days, present or absent major bleeding.

### Ethics and consent form

This study was submitted to and approved by the REC of the institution and will follow all the norms and precepts of the national and international resolutions, as described in resolution 466, of December 12, 2012, and complementary resolutions of the National Council of Health/Ministry of Health. This is a clinical trial in which interventions to be performed in both arms are considered physiological by the current literature, respecting the principle of clinical *equipoise*. All patients must have provided informed consent before randomization in this study. The informed consent form will be obtained by the principal researcher, who will be unaware of the allocation sequence. Such collection may be performed from the patient or legal guardian. All patients selected for transplantation will be included in the screening of the study; those eligible according to the inclusion and exclusion criteria will be informed about the protocol in a standardized way for deciding on participation, including information about the intervention and possible laboratory collections. The protocol will be submitted for publication in order to ensure methods transparency, and the final results will be disclosed to those involved and sent for publication under peer review, with a description of the degree of contribution of the authors.

### Study perspectives

According to the updated status on Clinicaltrials.gov, the study is currently in the randomization phase and has reached 67.8% of the required sample as of February 28, 2023. Maintaining the current randomization rate, the predicted end of inclusion will be at the end of the first half of 2023.

## COMMENTS

The present study was designed to evaluate a simple strategy with easy clinical application for a frequent, morbid and challenging outcome in liver transplantation. The decision for the primary outcome is based on biological plausibility, avoiding the previous scientific trend of seeking gross reductions in mortality. We understand that the single-center design may raise questions regarding the external validity of the outcomes, although we believe that the construction of evidence-based medicine occurs by the sum of the different efforts initiated in physiological and observational studies. The postoperative period following liver transplantation is an infrequent topic in randomized studies worldwide. We believe that the study presented here, with its simple and pragmatic description, may help to promote the current expansion of clinical trials in Brazilian intensive care.

The LIVER-PAM study presented here seeks to understand whether a target of higher (85 - 90mmHg) *versus* lower mean arterial pressure (65 - 70mmHg) for a period of 24 hours after admission to the intensive care unit for liver transplantation is sufficient to reduce the incidence of renal dysfunction in this scenario. Using a single-center, prospective, randomized, controlled and open-label model, we sought to answer a gap in the management of these patients, who are infrequently evaluated by randomized clinical trials. If our hypothesis proves to be true, we will contribute to the description of a potentially safe and inexpensive tool in the prevention of renal dysfunction following liver transplantation.

## APPENDIX 1 DEFINITIONS OF OUTCOMES

**Protocol:** Comparison between different mean arterial pressure targets in patients undergoing liver transplantation in an open-label, controlled, single-center, randomized clinical trial.

□ **Acute renal dysfunction**

Only patients with an increase in serum creatinine by 1.5 times the baseline value (last value prior to transplantation) will be considered to have renal dysfunction according to the adaptation of the KDIGO criterion referenced in the main text. For this, the diuresis criterion was ignored, as it can be difficult to interpret the real meaning of these data in the postoperative period and in cirrhotic patients. An increase of 1.5 times was used instead of the criterion of an increase of 0.3mg/dL because the calculated sample was based on the criterion of 1.5 times the baseline creatinine.^(1)^

□ **Death within 28 days**

Death within the first 28 days after liver transplantation.

□ **Retransplantation in 28 days**

Conduction of a new liver transplantation within the first 28 days after the initial transplantation.

□ **Renal replacement therapy in 7 days**

Administration of renal replacement therapy in the first 28 days after liver transplantation by any modality, prescribed by a specialist team of the institution itself, based on the classic indications (refractory hyperkalemia, refractory hypervolemia, refractory acidosis, refractory hypernatremia or uremia). Patients undergoing renal replacement therapy for ammonia clearance will not be considered.

□ **Surgical site infection**

Infection at the site of the surgical incision that appears to be related to the surgical procedure, manifesting as purulent secretion through a drain placed in the incision of the organ/cavity; organisms isolated from a positive culture of secretions or tissue of the organ/cavity obtained aseptically; presence of abscess or other evidence of infection, involving the organ/ cavity, identified in direct examination, during a new surgery, or through histocytopathological examination or imaging, which results in surgical approach and/or initiation of a new regimen of antibiotic therapy, as indicated together by the surgical and infectious diseases teams.

□ **Days alive and free of renal dysfunction in 7 days**

Number of days during the first 7 postoperative days that the patient was alive and did not meet the criteria for renal dysfunction consistent with the primary outcome

□ **Acute renal dysfunction according to urinary NGAL assessment**

Number of days during the first 7 postoperative days that the patient was alive and did not meet the following criteria for renal dysfunction: urinary NGAL value > 150ng/mL. This outcome will be assessed at admission and at 48 hours.^(2)^

□ **Primary graft dysfunction - initial poor function subtype at 7 days**

Development of one of the following laboratory criteria within the first 7 days after liver transplantation: bilirubin > 10mg/dL on the seventh day after transplantation; international normalized ratio > 1.6 on the seventh day after transplantation; alanine aminotransferase or aspartate aminotransferase > 2,000IU/L in any of the first 7 days after transplantation.^(3)^

□ **Primary allograft dysfunction - primarily nonfunctional subtype at 10 days (Broering)**

Need for retransplantation in the first 10 days after transplantation or death due to a nonfunctioning graft.^(3)^

□ **Major adverse kidney events in 28 days (MAKE28)**

The incidence of death, need for renal replacement therapy or persistent renal dysfunction at 28 days after liver transplantation.^(4)^

□ **Days alive and hospitalization-free in 28 days**

Number of days among the first 28 postoperative days in which the patient was alive and hospitalization-free.

□ **Major postoperative bleeding at 7 days**

Dichotomous outcome of bleeding that indicates, on the same day, the transfusion of more than two units of packed red blood cells or the need for surgical intervention indicated in the first 24 postoperative hours.

□ **Transfusion of blood components in 7 days**

Number of blood components transfused in 7 days separated into packed red blood cells (one unit equal to one bag), fresh frozen plasma (one unit equal to one bag), fibrinogen concentrate (one unit equal to 1g) and tranexamic acid (one unit equal to 1g).

□ **Arrhythmias requiring chemical or electrical therapy within 7 days**

Description in the prescription or medical records of antiarrhythmic or electrical cardioversion therapy for the first 7 days for treating abnormal rhythm. A maximum of one event per patient will be considered dichotomously, as the same event can be reentrant.
